# Applying Hybrid Lstm-Gru Model Based on Heterogeneous Data Sources for Traffic Speed Prediction in Urban Areas

**DOI:** 10.3390/s22093348

**Published:** 2022-04-27

**Authors:** Noureen Zafar, Irfan Ul Haq, Jawad-ur-Rehman Chughtai, Omair Shafiq

**Affiliations:** 1 Pakistan Institute of Engineering and Applied Sciences, Islamabad 44000, Pakistan; noureen_zafar@uaar.edu.pk (N.Z.); jawadchughtai@gmail.com (J.-u.-R.C.); 2 University Institute of Information Technology, Pir Mehr Ali Shah University of Arid Agriculture, Rawalpindi 46000, Pakistan; 3 School of Information Technology, Carleton University, Ottawa, ON K1S 5B6, Canada; OmairShafiq@cunet.carleton.ca

**Keywords:** IoT, LSTM, GRU, CNN, ITS

## Abstract

With the advent of the Internet of Things (IoT), it has become possible to have a variety of data sets generated through numerous types of sensors deployed across large urban areas, thus empowering the notion of smart cities. In smart cities, various types of sensors may fall into different administrative domains and may be accessible through exposed Application Program Interfaces (APIs). In such setups, for traffic prediction in Intelligent Transport Systems (ITS), one of the major prerequisites is the integration of heterogeneous data sources within a preprocessing data pipeline resulting into hybrid feature space. In this paper, we first present a comprehensive algorithm to integrate heterogeneous data obtained from sensors, services, and exogenous data sources into a hybrid spatial–temporal feature space. Following a rigorous exploratory data analysis, we apply a variety of deep learning algorithms specialized for time series geospatial data and perform a comparative analysis of Long Short-Term Memory (LSTM), Gated Recurrent Unit (GRU), Convolutional Neural Network (CNN), and their hybrid combinations. The hybrid LSTM–GRU model outperforms the rest with Root Mean Squared Error (*RMSE*) of 4.5 and Mean Absolute Percentage Error (*MAPE*) of 6.67%.

## 1. Introduction

Recently, the explosive growth of sensors, the internet, and huge data generation have provided new avenues of storage, execution, and implementation opportunities for IoT-based applications. Where the real-time availability of heterogeneous data generated from a huge number of sensors has brought about novel possibilities, a new set of challenges has also emerged, which is primarily related with the need of methodologies and capabilities to optimally harness the power of heterogeneity of data. Unveiling new interpretations by first integrating various forms of heterogeneous data and then applying intelligent algorithms to it is one of the most exciting possibilities of the notion of the Connected World [1].

Intelligent Transportation System (ITS) plays an enabling role in the realization of the concept of smart cities [2]. ITS has a huge demand to integrate data directly from various sensors, services built upon sensors, and from a variety of other exogenous data sources. ITS heavily relies on the power of IoT to build multisource, multisensor, and multimodel service systems for the prediction of traffic speed [3,4].

Major challenges faced during the activities of the data integration pipeline include sparsity handling, anomaly detection and rectification, normalization, map matching, and data resolution. The resolution quality of the fused and integrated traffic data plays a significant role when prediction algorithms are applied to it. The spatial temporal nature of traffic data defines the two corresponding facets of its resolution on GIS maps. Spatial resolution identifies the length of the road segment on which prediction is computed whereas the temporal resolution means the minimum time interval during which the prediction is made. The spatial temporal structure is used to integrate traffic data from different sources, e.g., Floating Car Data and Google in our case. Data elements from different sources at specific road segments during a specific time interval are fused together, thus adding value to accuracy. Necessary transformations from Estimated Time of Arrival (ETA) to speed or vice versa are performed before the fusion. This may result in spatiotemporal gaps due to the varying sparsity of data sources that need to be addressed. Data from the exogenous sources, e.g., weather, holidays, or peak hours, are also weaved upon the same spatial temporal framework in a similar fashion.

In this research, a detailed workflow is developed for the data integration pipeline that transforms raw data extracted from different heterogeneous data sources, passing through various preprocessing, and integration activities finally resulting into an integrated hybrid feature space. Open Street Maps (OSM) was selected as the GIS mapping technology. The integrated data are mapped on the road segments between every two adjacent OSM nodes within the time intervals of 15 min for the whole 24 h of the day. Keeping in mind the spatial temporal nature of the data, machine learning and deep learning algorithms specialized for time series data were applied, and their results were compared.

The main contributions of the paper can be summarized as follows:

- We integrated the heterogeneous data sources of Intelligent transportation systems for data collected from a particular city in Pakistan and built the hybrid LSTM-GRU model.
- We predicted the traffic speed on the basis of heterogeneous traffic data sources including exogenous data sources, e.g., weather, event and, peak hours.
- The Hybrid LSTM-GRU model has been applied on time intervals varying from 15 min to 1 h and the effectiveness of the model has been evaluated.

The remainder of the paper is organized as follows: Section 2 presents the related work. Section 3 presents the proposed methodology based on Hybrid LSTM–GRU model. Section 4 provides the results and discussions. Section 5 concludes the paper and identifies the future research direction.

## 2. Related Work

The related work was organized in context with the integration of heterogeneous data sources obtained from a variety of sensors leading to deep learning techniques applied on the spatial–temporal traffic data.

Salanova Grau et al. [5] presented a novel auto-DL approach for tuning the 145 errors and 22 warnings hyperparameter of the LSTM model in order to reduce time consumption. However, the author worked only on temporal features, not spatial features, and also did not focus on multidata sources.

Tang et al. [6] improved the fuzzy neural network model in order to enhance the learning capability of the model. The proposed model consists of a supervised and unsupervised learning process. In unsupervised learning, a k-means is used to partition input samples, and in the case of supervised learning, a weighted recursive least squares estimator is used for the optimization of hyperparameters. However, spatial patterns were not considered, while periodic features were extracted from traffic flow data. The authors did not deal with heterogeneous data sources of the arterial road network.

Ma, Tao, Wang, and Yu [7] proposed the novel approach LSTM-NN to overcome the issue of backpropagated error decay and automatically determine the optimal time window for the time series data set. This means the short-term prediction of speed deals with NN and long-term prediction of speed deals with LSTM. The authors used microwave detector data for the prediction of speed. However, they have only addressed the temporal features. There was a need to address the spatial features as well as a need to investigate with different data aggregated levels that would act as an additional input and contribute towards the prediction of speed in a more accurate consistent manner. Salavona Grau et al. [1] proposed the framework that collects data from conventional(cameras, radars, and loops) as well as innovative (FCD and Bluetooth devices) technologies and analyzes the ETA and traffic flow on road networks. They are focused on data collection, filtration, and fusion of big data. They analyzed the conventional and innovative types of data sources with the help of statistical approaches such as correlation coefficients, R-square, absolute value, and percentages ranges. However, there is a need to check the spatial–temporal features of big data on the road networks with the help of a machine and deep learning models that are specially proposed to handle the time series and spatial patterns. Bratsas et al. [8] applied different machine learning models and figured out the forecasting effectiveness of the machine learning model on randomly selected dates, randomly selected roads with duration over eight consecutive 15 min intervals, and the whole day. The experiments show that SVR performed well with stable conditions and MPL performed well on greater variations. However, the paper does not analyze the specialized deep learning models that are designed to deal with spatial–temporal trends of the data.

Yang et al. [9] present the effects of heterogeneous data sources (parking meter transactions, traffic conditions, and weather conditions) and spatial–temporal nature of data on the parking occupancy by linking Graph CNN and LSTM deep learning models. However, the authors did not incorporate ETA, event, holiday, calendar, OSM, and count data sources in their work. The authors focused on single-scale occupancy prediction rather than multiple scales, i.e., parking meters to the level of aggregated zones.

In order to cope with chronological deviations for traffic forecasting, spatial–temporal patterns have been extensively addressed [10,11,12,13,14,15,16,17,18,19,20,21,22,23] using dynamic tensor completion method, LSTM, temporal graph convolutional network, multivariate regression model, GraphCNN-LSTM model, attention graph convolutional sequence-to-sequence model, and spatial–temporal residual graph attention network. The authors, however, have not considered the effects of heterogeneous and nonrecurrent events data sources for forecasting road traffic.

Zhang et al. [24] proposed the deep learning-based multitask learning model to predict traffic speed on the road networks. The authors used the hybrid approach to increase the performance of the proposed model on taxis GPS data. They extracted the spatial–temporal patterns with the help of the nonlinear Granger causality analysis method, and for hyperparameter tuning, they used the Bayesian optimization technique. However, the authors did not consider other modes of traffic and their influence on the prediction of speed.

Kong et al. [25] proposed the recommendation system of intelligent traffic based on LSTM deep learning model. However, they ignored the positive impact of performance by using a multimodel on intelligent traffic information and also did not make a comparison of the famous time-recurrent neural network models, e.g., CNN [26], GRU, and hybrid models.

Authors in [27,28,29] used hybrid graph convolutional neural networks, CNN-LSTM, and LSTM-NN models on real-time traffic speed. The hybrid model produced promising results; however, other data sources related to road traffic that have directly or indirectly influenced the prediction of speed on road network were not taken into account.

Authors in [30,31,32] used k-means clustering, PCA, SOM, SVM, and SVR for prediction of traffic speed on regular or irregular intervals. However, their prediction models can be improved with a fusion of nonrecurrent events(e.g., calendar, special events, accidents, and weather) and traffic flow analysis.

Li et al. [33] proposed the transfer-learning model to address the missing data, data insufficiency, and mitigate model overfitting problems and stack LSTM for considering the time series patterns. However, the authors did not analyze the exogenous data sources, traffic types, and different traffic modes data sets. Hybrid feature space may be helpful for the construction of rules for applying transfer learning on the specific area while considering spatial factors.

Mena-Oreja et al. [34] discussed the formation of congestion by using state-of-the-art error recurrent and deep learning models. The author conducted a survey in the field of transportation and identified which deep learning and error recurrent models are helpful while considering spatial–temporal factors and other traffic conditions. The author applied the error recurrent and deep learning models on real traffic data sets in order to generate the common benchmark under traffic congestion conditions and demonstrated that the error recurrent model shows better accuracy as compared to deep learning models. However, the authors do not focus on the impact of statistical and ensemble deep and machine learning models on traffic congestion conditions using real traffic data sets and also do not discus hybrid feature space and its impact on traffic congestion prediction.

Ren et al. [2] propose the hybrid integrated–DL model in order to capture both spatial–temporal dependencies on the prediction of citywide spatial–temporal flow volume. The authors proposed the hybrid LSTM and ResNet model in order to deal with spatial–temporal effects on traffic volume. However, the authors proposed that the model shows large prediction error in sparse spatial areas and sleeping hours. The authors do not focus on the applicability of other types of flows, e.g., passenger flow, bike flow, etc.

Yu et al. [35] introduced the piece-wise correlation function and Jenks clustering method with dynamic programming to fix relationship of segment intervals. They considered heterogeneous data sources, e.g., speed, traffic flow, density, and road occupancy for short-term speed prediction on 5 and 10 min time intervals. They prepared the results on the basis of only three days, i.e., 1 February to 3 February 2015 (from Sunday to Tuesday). They did not discuss the effects of the weekdays and nonweekdays on road traffic. The amount of data is very low, and it is difficult to explore the correlation function across the whole week.

Liu et al. [36] used attention CNN to forecast traffic speed. They used 29,952 records as a training set and 5760 records as the test set. The amount of data is very low due to coverage of single road and difficult to explore traffic trends on their adjacent roads. In an earlier work [37], we built a solution that used traffic and weather data to predict traffic congestion using Estimated Time of Arrival (ETA).

We proposed a hybrid LSTM–GRU approach on heterogeneous data sources which comprises 7,343,362 records of September 2020. We made a comprehensive comparison among famous time-recurrent neural network advanced deep learning models, e.g., CNN, GRU, and their combinations.

## 3. Proposed Methodology Based on Hybrid LSTM–GRU Model

We obtained Floating Car data from a regional trackers’ company. A data integration pipe-line was developed to clean, preprocess, and integrate this data with other data sources. Data integration pipe-line caters data pertaining to FCD, Google, weather, peak hour, holiday, and OSM data sources. We addressed the issues related to the FCD data sources—some of which include zero speed adjustments, outlier removal, and map matching. A feature in FCD data provides the reason for a signal generation from the tracker. The reason may be regular time interval, ignition on/off, turn, etc. A map-matching technique was used for node correction whereas threshold value, parking info, and ignition on/off details were used for zero speed adjustment. Finally, all the data sources were aggregated on the basis of wayid attribute of OSM and time attribute at a regular interval of 15 min. This was followed by the normalization of road speed through Permissible Speed Limit (SPL) attribute and Speed Performance Index (*SPI*).

### 3.1. Data Sources

Data from the following data sources were obtained and integrated.

#### 3.1.1. FCD Data Source

FCD data were obtained from a regional tracker company. The data set contained events generated by 2895 unique tracker ids for the whole month of September 2020. The tracker units were mounted with the sensors GSM Modem(Quectel M95) and GPS Chipset(U-blox EVA-M8M). The key features of Floating Car Data (FCD) include latitude, longitude, date time, address, location, direction, speed, reason, and unit id. We faced multiple issues related to data preparation of FCD data source. The following issues were identified in FCD data:

- There was an off-road mapping of cars. This could be due to two reasons. Either the car appears offroad because of inherent GPS error or because the car was actually parked somewhere off the road.
- A large number of speed values generated by trackers were zero. This again could be due to two reasons: either the car is parked or stuck in congestion. The congestion data needed to be distinguished from the data related to the parked cars.
- There was duplication of tuples.
- There are missing values causing spatial sparsity. This is because the FCD does not cover all segments of roads of the road network.

The issues pertaining to the missing values and off-road mapping of cars are resolved through the map-matching process described in Algorithm 1. When the nodes are placed correctly on the map, the in-between missing values can be suggested. The zero speed issue is resolved through the zero speed adjustment section described in Algorithm 1. Outliers are identified and rectified through the max speed attribute obtained from OSM. Any speed exceeding the max speed on a segment of the road is replaced with the max speed value. Speed Performance Index is used for the normalization of data.
**Algorithm 1** Preprocessing and Data Integration Algo1:Read coordinate point [latitude, longitude] from data2:Initialize nodes-seg with empty start-point and end-point3:**for** t = 1 to m **do**4:    Initialize way-id to zero5:    nodes-seg[“start-point”,“end-point”]:=get_nearest_seg(latitude,longitude)6:    **if** nodes-seg [“start-point”] is zero **then**7:        Check end-point value and update the start-point value with previous node value8:    **else**9:        nodes-seg [“start-point”] and nodes-seg [“end-point”] represents to same road points10:    **end if**11:**end for**12:Update way-id13:data.append(way-id)14:Update data15:take input records from data16:F = 017:**for** t = 1 to n **do**18:    Initialize speed = data[“FCD-speed”]19:    Initialize elapsed time = data[“FCD-elapsed-time”]20:    **if** reason is “ignition-ON” **then**21:        F = 122:        **if** next reason is “ignition-OFF” **then**23:           **if** speed is zero **then**24:               **if** elapsed time >threshold **then**25:                   continue F = 126:               **end if**27:               **if** next speed is zero **then**28:                   F = 029:                   delete record30:               **else**31:                   keep record32:               **end if**33:           **else**34:               F = 035:               keep record36:           **end if**37:        **end if**38:        remove record39:    **end if**40:**end for**41:Initialize the data to empty42:**for** each seg **do**43:    **for** each agg-min each day **do**44:        Compute avg-speed = $\frac{1}{n}\sum x_{i}$45:        Initialize s = length of the segment46:        Compute eta = L ÷ avg-speed47:        data[“avg-speed”].append(“avg-speed”)48:        data[“eta”].append(“eta”)49:    **end for**50:    **if** current_day is “Sunday” or “Saturday” **then**51:        data[“holiday”] = 152:    **else**53:        data[“holiday”] = 054:    **end if**55:    data[“weather”] = get weather of the current day with respect to current location56:**end for**

#### 3.1.2. ETA Data Source

We decided to obtain Google Map’s data from more than 500 points of interest on important roads of Islamabad. We acquired data by sending start and endpoints to Google Maps API. The Google data source is an authentic data source and provides an estimated time of arrival information. Data obtained from Google can be easily mapped on OSM Maps. The key features of Google data are Source Latitude, Source Longitude, Max Speed, Date, Time, Destination Latitude, Destination Longitude, and Estimated Time Arrival.

#### 3.1.3. OSM Data Source

The road network attributes were fetched from OSM’s Turbo Overpass API. It provides the tags of Islamabad which include start node, end node, highway types, max speed, min speed, way_id, and max length. Max speed is a feature of special importance that is utilized in the normalization of data and outlier identification and removal. Some roads do not have a max speed feature associated with them; therefore, we had to insert it manually.

#### 3.1.4. Calendar Data Source

In order to identify the effect of traffic on holidays at a particular location, we need a calendar data source. Behaviors and patterns of traffic are highly dependent on holiday data. The calendar data source features include DateTime, Name, and Type.

#### 3.1.5. Weather Data Source

Real-time data source is gathered from Yahoo and Dark Sky API on the basis of time and latitude and longitude. Table 1 shows the most relevant features of the proposed hybrid model. The maxspeed-real attribute is used to detect anomalies and normalized the speed.

From feature engineering process, we exploited the input feature set and then applied machine and deep learning models that are capable enough to consider spatial–temporal effects on transportation data sources. We also applied hybrid approaches such as LSTM-GRU, GRU-LSTM, CNN-LSTM, and CNN-GRU as these hybrid approaches enhance the performance of models and provide more accurate prediction of the speed of a specific road at a specific time.

(1) $$SPI=(S_{i}^{t}/S_{PL})*100$$

### 3.2. Data Integration Pipeline

In the Integration Pipeline, we performed the transformation on FCD and Google data set. The map-matching process was used to obtain the data in spatial format. Hence, the first transformation in the pipeline is the map matching.

**Figure 1:**
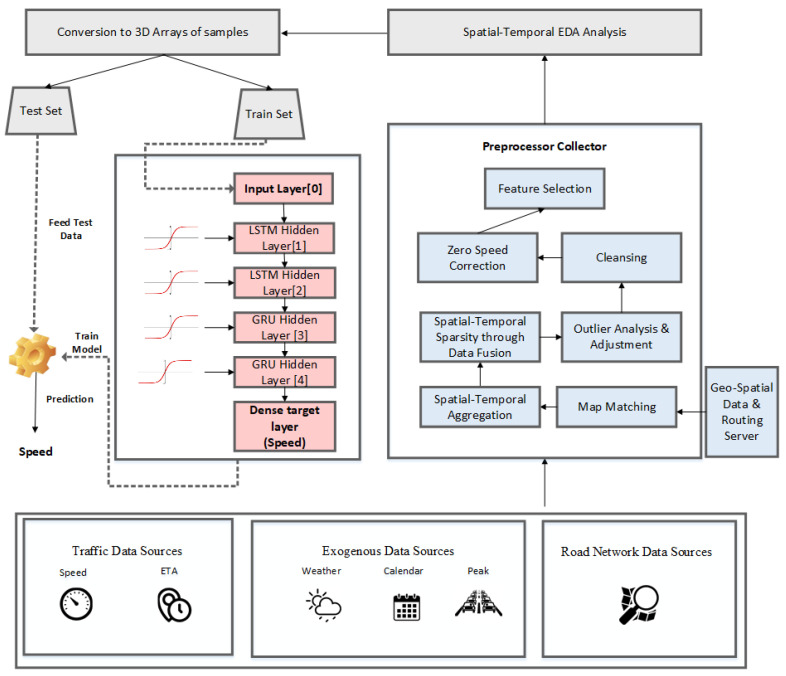
Urban traffic speed prediction based on hybrid LSTM–GRU model.

Figure 1 illustrates the data integration procedure. Data are collected from different data sources such as Google, tracking company, osm road, weather data, holiday data, and peak hour data. In addition to the map-matching procedure, the following activities were required for data transformation before the machine learning algorithms could be applied:

- Map matching of GPS points
- Handling the abnormal behavior of data
- Data generalization and transformation
- Calculating the average speed of road section.

In the Google data set, the data points needed to be mapped on the OSM nodes. In this way, we could divide long roads in smaller segments with each segment marked by two adjacent OSM nodes. For this purpose, the nearest API of OSRM was again used. We verified the mapping results by visualizing the nodes on OSM maps. The mapping of FCD data and Google traffic data on OSM nodes provided a mechanism to spatially unify both traffic data. For temporal aggregation, the data points of both data sources were aggregated for every 15 min for all the road segments defined on the OSM road network. The integrated data were then merged with holiday data based on the date field. The purpose of the abovementioned integration was to encounter the special effects of congestion on working hours and weekends during holidays. These merged data are further integrated with road attributes on the basis of wayid. Furthermore, to handle the environmental effects, we combined these integrated data with only those weather data parameters that affect the behavior of the traffic, i.e., rainy, visibility, etc.

Algorithm 1 represents the procedure of map matching. The data contains the coordinate points with the latitude and longitude of the located geographical positions. Firstly, these coordinates were sent to the nearest API of OSRM server to obtain the pair of nodes of the segment containing the location of the driving vehicle that had already been determined from different sources. This was followed by the ordering of nodes on OSM maps to identify whether the road is incoming or outgoing. Occasionally, OSRM nearest API returns zero value in place of start node that might be due to multiple options for the nearest start node owing to the junctions on roads. For zero values, the end-node value was traced back on the OSM road information, and the immediate previous node was assigned to the start-node value.

### 3.3. Model Selection

We have applied the existing machine learning, classical deep learning and advanced deep learning models on the integrated traffic data. We have applied the well-known models such as XGBoost [38] (with Decision Tree [39] as weak learner), Linear Regression [40], K-Nearest Neigbor (KNN) [41], Multilayer Perceptron (MLP) based on MLPRegressor implementation in sklearn library (available at https://scikit-learn.org, accessed on 1 October 2021) [8], Artificial Neural Network (ANN) based on KerasRegressor implementation in keras library (available at https://keras.io, accessed on 1 October 2021) [1], Long Short Term Memory (LSTM) [42], Gated Recurrent Unit (GRU) [43], Convolutional Neural Networks (CNN) [44], and well-known possible combinations of the models such as LSTM-CNN, CNN-LSTM, CNN-GRU, GRU-CNN, GRU-LSTM and LSTM-GRU models. Due to the spatial-temporal nature of the data models based on LSTM and GRU techniques generated better results. The Hybrid LSTM-GRU model has produced the most promising results.

In the following subsections we briefly describe the existing and well-known LSTM and GRU techniques and then move on to describe our hybrid model based on combining these existing techniques.

#### 3.3.1. LSTM

Long Short Term Memory (LSTM) [42] is a variant of recurrent neural network (RNN) [5,45]. It is specialized for time series data. A generalized LSTM unit consists of three gates (i.e., input, output and a forget) and a cell. Cells are used to memorize the values of data and flow the information to output and forget gate. It is used to address vanishing gradient problem.

#### 3.3.2. GRU

Gated Recurrent Unit (GRU) [43] is an advanced and more improved version of LSTM. It is also the type of recurrent neural network. It uses less hyper parameters because of reset gate and update gate as contrast to three gates of LSTM. Update gate and reset gate are basically vectors and are used to decide which information should be passed to the output.

#### 3.3.3. Hybrid LSTM-GRU Model Description

Since, our dataset is time series and regression problem, we generated the results by using both classical as well as deep learning techniques. Our hybrid approach techniques yielded the low *RMSE* and is thus more effective as compared to classical regression techniques e.g., KNN, XGBoost, Linear Regressor, ANN and MLP. In our hybrid LSTM-GRU model, we first applied LSTM. LSTM is used to tackled the problem of vanishing gradient in backpropagation. LSTM contain three gates e.g., input gate ($i_{g}$), forget gate ($f_{g}$) and output gate ($o_{g}$). Gates are used to store information in memory. It stores the information in analog format. These gates are element-wise multiplied by sigmoid function ranges between 0–1. If the value of the gate is zero then this information is ignored or discarded else remained in memory. Tanh [46] is a well-known non-linear activation function and ranges between −1 to +1. In order to avoid information fading, a second derivative is used. The sigmoid function [47] is also well-known as a non-linear activation function. A sigmoid function contains values between 0 to 1. It is basically used to suggest which information should stay or drop from memory units known as gates. The mathematical Equations of input gate ($i_{g}$), forget gate ($f_{g}$) and output gate ($o_{g}$) are taken and adapted from the literature and are explained in Equations (2)–(4). Whereas, GRU contains two gates e.g., update gate ($u_{g}$) and reset gate ($r_{g}$). The output of the LSTM was passed to GRU during this approach. $x_{i}^{t}$ is the input feature set that contains hybrid feature space(start-node, end-node, way-id, day, hour, agg-minutes, quarter, holiday, peakhour, mazspeed-real) at specific time and location. The aggregate speed is the target or output label, similar to [48] in which authors used LSTM and Bi-Directional LSTM Models to predict stock price.

The details and equations presented are taken and adapted from the literature such as [42,43].

Input Gate: $i_{g}\to represents\;input\;gate$
Forget Gate: $f_{g}\to represents\;forget\;gate$
Output Gate: $o_{g}\to represents\;output\;gate$
Update Gate: $u_{g}\to represents\;update\;gate$
Reset Gate: $r_{g}\to represents\;reset\;gate$

σ→representssigmoidfunction

$w_{x}\to represents\;weight\;for\;the\;respective\;gate\;\left(x\right)$

$h_{t-1}\to output\;of\;the\;previous\;lstm\;block\;at\;timestamp\;t-1$

$x_{t}\to input\;at\;current\;timestamp$

$b_{x}\to biases\;for\;the\;respective\;gates\;\left(x\right)$

Cell Output: $C_{t}\to memory\;at\;timestamp\;\left(t\right)$
Cell Input: $∼C_{t}\to represents\;candidate\;for\;memory\;at\;timestamp\;\left(t\right)$

The Cell Input state is $∼C_{t}$, Cell Output state is $C_{t}$, and LSTM consists of three gates $i_{g}$, $f_{g}$, and $o_{g}$. GRU consists of two gates $u_{g}$ and $r_{g}$. The hidden layers of LSTM–GRU model are $∼C_{t}$, $∼h_{t}$, and $h_{t}$. The weights of LSTM are $w_{i}$, $w_{f}$, $w_{o}$, and $w_{c}$. The weights of GRU are $w_{u}$, $w_{r}$, $w_{o}$, and $w_{C_{t}}$. LSTM–GRU model have biases $b_{i}$, $b_{f}$, $b_{o}$, and $b_{c}$. tanh is known as the hyperbolic tangent function. The ratio of the hyperbolic sine and corresponding hyperbolic cosine functions is defined in terms of tanh function. The scalar products of two vectors are represented as ∘.

When $x_{t}$ is passed to the input network unit, it is multiplied by its own weight ($w_{i}$), and $h_{t-1}$ is also multiplied by its own weight ($w_{i}$) and then added the bias ($b_{i}$). A $h_{t-1}$ holds the information of previous units t−1. It passes to the sigmoid function and converts values between 0 and 1 and updates the status of the cell. The details and equations presented are taken and adapted from the literature such as [42,48,49].

(2) $$i_{g}=\sigma \left(w_{i}\left[h_{t-1},x_{t}\right]+b_{i}\right)$$

(3) $$f_{g}=\sigma \left(w_{f}\left[h_{t-1},x_{i}\right]+b_{f}\right)$$

(4) $$o_{g}=\sigma \left(w_{o}\left[h_{t-1},x_{t}\right]+b_{o}\right)$$

Equations (5) and (6) describes how to produce the result between 0 and 1 using sigmoid activation function. $∼C_{t}$ and $C_{t}$ are used to decide what information is kept in memory and what information is forgotten. $∼C_{t}$ is multiplied by the tanh function and decides which value is more significant.

(5) $$∼C_{t}=tanh\left(w_{c}\left[h_{t-1},x_{t}\right]+b_{c}\right)$$

(6) $$C_{t}=f_{t}*C_{t-1}+i_{t}*∼C_{t}$$

The details and equations presented are taken and adapted from the literature such as [43,48]. Equations (7) and (8) explain that $C_{t}$ is passed as an input to the first layer of GRU ($u_{g}$), whereas $u_{g}$ and $h_{t-1}$ are multiplied to weight and this information is forwarded to reset gate ($r_{g}$).

(7) $$u_{g}=\sigma \left(w_{u}\left[C_{t}\right]+w_{u}\left[h_{t-1}\right]\right)$$

(8) $$r_{g}=\sigma \left(w_{r}\left[C_{t}\right]+w_{r}\left[h_{t-1}\right]\right)$$

$h_{t}$ decides information to be kept. The stayed information is then attached to the output layer. Same layer contains tanh as an activation function that is used to predict road traffic speed at specific time and location. This is also discussed in Equations (9)–(11). We used adam as an optimizer and mean squared as loss function in this regression problem.

(9) $$∼h_{t}=tanh\left(w_{C_{t}}+r_{g}\circ w_{C_{t}}\left[h_{t-1}\right]\right)$$

(10) $$h_{t}=u_{g}\circ h_{t-1}+\left(1-u_{g}\right)\circ ∼h_{t}$$

(11) $$h_{t}=o_{g}*tanh\left(h^{t}\right)$$

In this study, the proposed LSTM–GRU model was applied to the data collected from Google and FCD which comprises 7,343,362 records of September 2020. The traffic condition data were captured every fifteen minutes of arterial roads in Islamabad, Pakistan. The proposed stacked LSTM–GRU architecture consisted of four hidden layers with 256 hidden units each. tanh was used as an activation function in all hidden layers. In the dense layer, we used one unit. The linear activation function was used in the output layer. We employed holdout crossvalidation to split data set into training and test sets. Then, we trained the model on the training data set by a batch learning approach using batch size of 512. This was followed by checking generalization of model on test data set. To evaluate the performance of the proposed deep architecture, we adopted *RMSE*, *MAE*, and *MAPE* as performance measures. The final configuration of the proposed LSTM–GRU model is summarized in Table 2. In the proposed model LSTM–GRU, we used four hidden layers (two for LSTM and the rest for GRU). We tested various configurations of hidden units, i.e., 4, 16, 32, 64, 128, and 256. We achieved the optimal results with 256 hidden units. Likewise, tanh activation function in the hidden layers and linear activation function in the output layer gives optimal results. Similarly, we varied the number of epochs from 5 to 50 and choose 10 as the optimal value. Moreover, we used batch size 512, learning rate 0.001, and loss function adam as the optimal parameter values.

#### 3.3.4. Performance Measures for Proposed Hybrid LSTM–GRU Model

Well-known and existing performance evaluation metrics are used to judge or measure the model performance and pattern. It also indicates the best model in order to achieve the output label performance. To evaluate the solution, we have used the existing performance evaluation metrics [50] such as *RMSE* (Root Mean Square Error), *MAPE* (Mean Absolute Percentage Error) and *MAE* (Mean Absolute Error).

The details and equations for the existing performance evaluation metrics presented below are taken and adapted from the literature such as [50,51,52]. *RMSE* is also known as RMSD ( root-mean-square deviation). It is the square root of the mean squared difference between desired output and predicted output. The same is explained in Equation (12).

(12) $$RMSE=\sqrt{(\sum_{i}{\left(Y_{dsi}-Y_{psi}\right)}^{2}/number\;of\;observations)}$$

where,

$Y_{dsi}$ = desired speed
$Y_{psi}$ = predicted speed
*n* = number of observations

*MAPE* (mean-absolute-percentage-error) is robust to large outliers. It eliminates the scaling factor and explains the error in the form of percentage. The formula of *MAPE* is explained in Equation (13).

(13) $$MAPE=median(|\left(Y_{dsi}-Y_{psi}\right)/Y_{dsi}|)$$

In our scenario, *MAE* (Mean-Absolute-Error) is used to define how far our predicted output speed is from the desired output speed. Mathematically, we can explain this from the following Equation (14).

(14) $$MAE=1/n\sum_{i}\left|\left(Y_{dsi}-Y_{psi}\right)\right|$$

## 4. Results and Discussion

In this study, we worked on the regression data set with the multitimestamp and single label. Some features related to traffic patterns were derived in each segments at specific interval of time such as minimum estimated time and maximum speed per segment from integrated data.

Data were captured at 15 min time resolution and taking the space resolution less than or equal to 1 km.

### 4.1. Exploratory Data Analysis

We analyzed the influence of weekdays and weekend on traffic patterns and speed fluctuation. Speed Performance Index (*SPI*) [53] is a derived feature and calculated by using the following formula in the Equation (15):

(15) $$SPI=(\left(V_{O}\right)/V_{m})*100$$

where,

$V_{O}$ = *the current speed for the road segment;*
$V_{m}$ = *the permissible max speed for the road segment.*

*SPI* also provides a normalized expected speed which prevents having extreme values.

**Figure 2:**
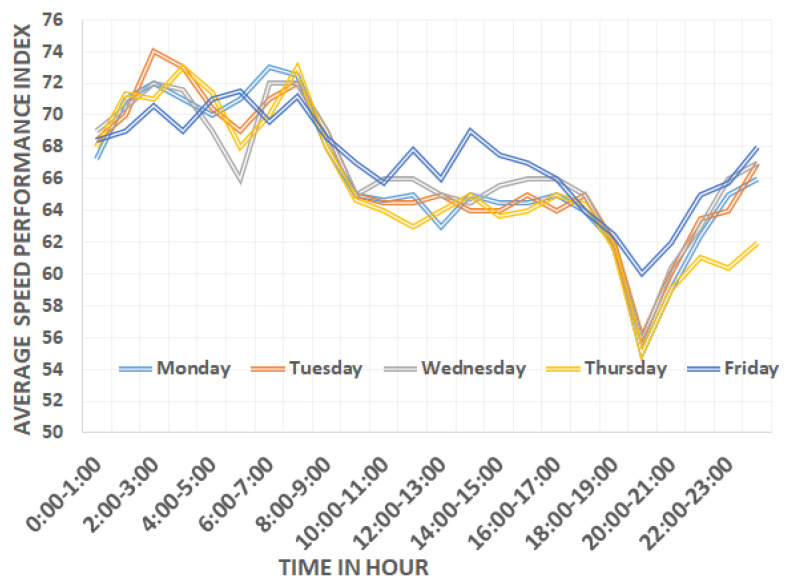
Speed Performance index variation on weekdays.

Figure 2 presents the average speed performance index (*SPI*) vs. time of day on weekdays. Different working days, i.e., Monday to Thursday, are shown with different color lines. In Figure 2, it can be visualized that there is a major change in the *SPI* over different hours of the day. In the morning rush hours (5:00–6:00 a.m., 9:00–10:00 a.m.,12:00–13:00 p.m.), the *SPI* is 66%, 64.6%, and 63%, respectively, which is higher than the average of morning hours. During the evening rush hour (around 8:00–9:00 p.m.), the *SPI* is 55%. The different trend of the *SPI* in the different time slots is not only compelling for model training but also in taking the average of the *SPI* for each time slot and for the prediction of speed on the basis of historical data. Various time slots including 5:00–6:00 a.m., 9:00–10:00 a.m., 12:00–13:00 p.m., and 8:00–9:00 p.m. show high traffic congestion. Friday’s *SPI* trend is different from other working days. On Friday, during the morning rush hour (10:00 a.m.–12:00 p.m.), the *SPI* is 65.7%, which is higher than the average of morning hours. Friday traffic is different from other weekdays due to the Friday prayer, which is offered during 1–2 p.m. During the evening rush hour (9:00–10:00 p.m.), *SPI* is 60%, which is higher than all the time of day.

**Figure 3:**
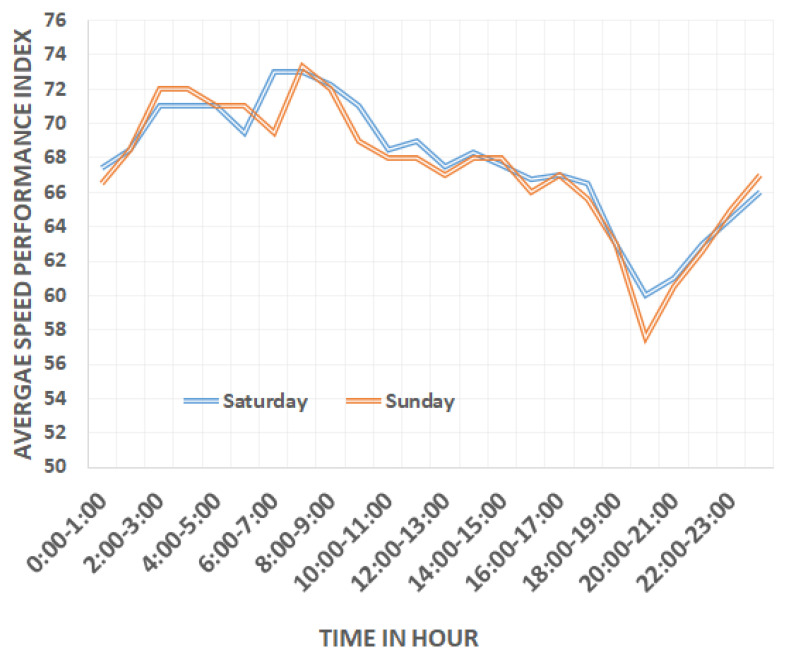
Speed performance index variation on weekends.

*SPI* variations on weekends are addressed in Figure 3. This figure depicts that weekend *SPI* trend is extremely opposite from weekdays *SPI*. On Saturday, rush hours are 10:00 a.m.–1:00 p.m. in the morning and 3:00–4:00 p.m., 9:00–10:00 p.m. in the evening, whereas on Sunday, rush hours are 12:00 p.m. to 1:00 p.m. in the morning and in the evening, 9:00 p.m. to 10:00 p.m., respectively.

### 4.2. Feature Selection

Feature selection is a method that was used to explore the most relevant feature set by using correlation. The correlation of each feature was calculated and compared with the target variable. The correlation ranged between −1 and +1. +1 shows the positive correlation or perfect correlation, whereas −1 shows negative correlation and zero value means nonexistence of correlation. Basically, we were dealing with numeric input and target features. As the most relevant and famous techniques that deal with numeric data are correlation feature selection and mutual feature selection techniques, we used the same ones in our study.

#### 4.2.1. Correlation Feature Selection Technique

Correlation is a statistical measure used to identify how two features changed together. We used Pearson correlation coefficient(PCC) [54] in our scenario.

Equation (16) explains that PCC is a standard measure of linear correlation between the two features. The formula of PCC is the ratio between the covariance of two features divided by their standard deviations. It deals between normalized data that range between −1 and +1.

V[:,j] = feature of $V_{j}$ in hybrid data set.
*j* = is a variable.
mean(V[:,j]) = mean of the $V_{j}$ feature of hybrid data set.
W = feature of W in hybrid data set.
$mean_{W}$ = mean of the W feature of hybrid data set.
std(V[:,J]= Standard deviation of V[:,j].
std(W) = Standard deviation of W.

(16) $$\left(\left(V\left[:,j\right]-mean\left(V\left[:,j\right]\right)\right)*\left(W-mean_{W}\right)\right)/\left(std\left(V\left[:,J\right]\right)*std\left(W\right)\right)$$

Table 3 depicts that start-node, way-id, hour, peak hour, and max speed-real are most relevant features as they are positively correlated, because in transportation domain start-node, way-id shows spatial impact, whereas hour and peak hour show temporal impact. Max speed-real shows the permissible speed of the road. We selected the 9 most correlated features from 29 combined features of all heterogeneous data sources. The correlation shows how all features are close to the target feature.

#### 4.2.2. Mutual Information Regression Feature Selection Technique

**Figure 4:**
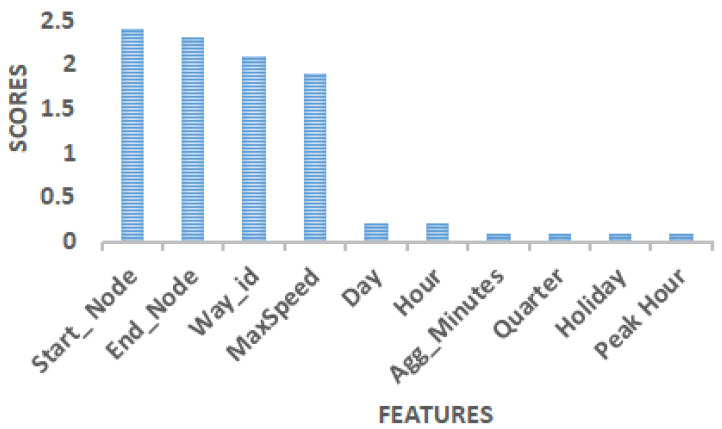
Feature selection using mutual information regression feature selection technique.

The mutual information feature selection technique is a method that works on information gain [55]. It works on the decision tree principle and calculates the entropy of each feature by calculating the information gain of each feature. Entropy helps the decision tree draw boundaries and measures disorder and uncertainty in the available data set. The most relevant feature has the highest information gain. Figure 4 indicates that start-node, end-node, way-id, and max speed have the highest information gain and are therefore the most relevant feature set.

#### 4.2.3. Heat Map of Hybrid Feature Space

**Figure 5:**
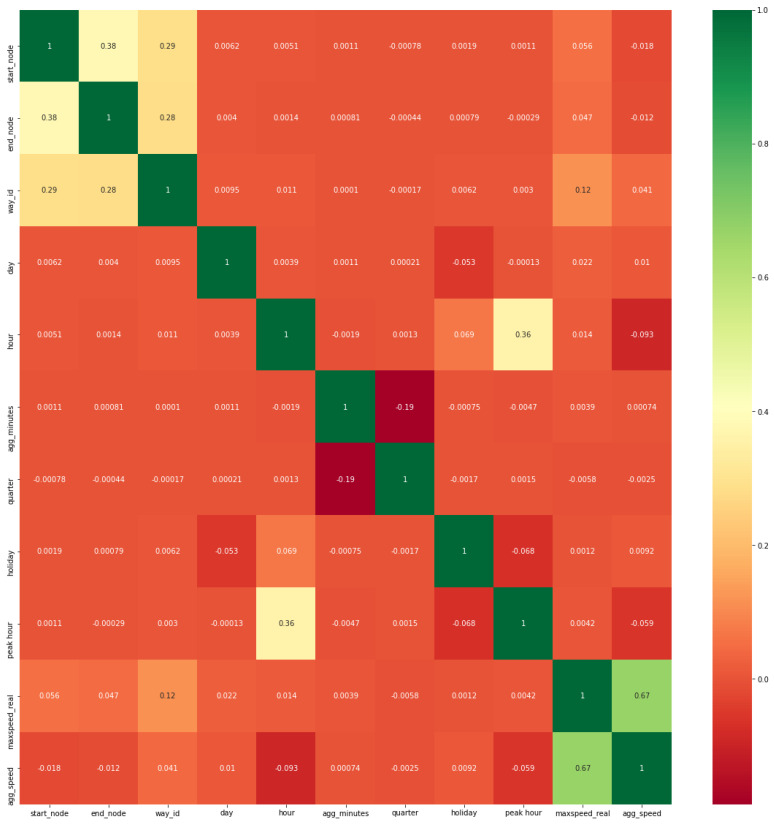
Heat map of hybrid feature space.

**Figure 6:**
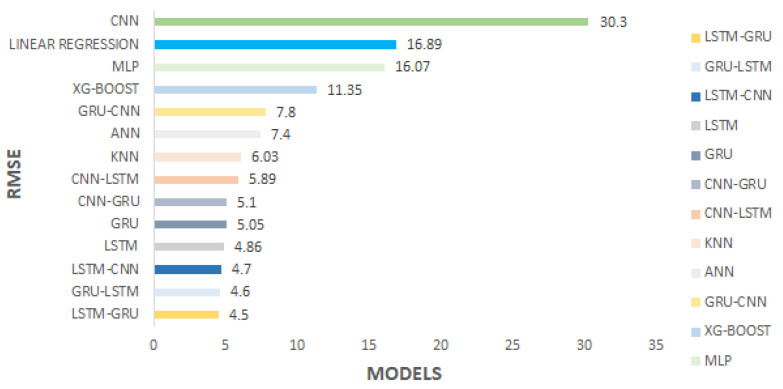
Evaluation metrics of prediction on test data.

Heat Map is a visualization style for analyzing the intensity, density, patterns, outliers, and variance of the feature set. It provides a correlation among all features. Figure 5 shows that maxspeed-real have the highest correlation with target agg-speed followed by way-id, day, and holiday features among the other heterogeneous features. Here, maxspeed-real denotes the permissible road speed limit. It helps to identify outliers and trends and patterns of road speed. In this way, we can tackle the missing speed and also normalize the data set in order to predict the true speed of a specific road. Figure 6 depicts that LSTM–GRU yields the lowest *RMSE*, i.e., 4.5 as compared to deep learning technique LSTM with a *RMSE* yield of 4.86 and classical regression techniques such as KNN with an *RMSE* yield of 6.03. Because the LSTM–GRU model handles both spatial–temporal effects, LSTM–GRU is specialized in time series data set. LSTM contains the temporal effects, and GRU contains the spatial effects. Because we have worked on transport data set and in the domain of transportation prediction of speed highly depends on specific time and location.

### 4.3. Hybrid LSTM–GRU Results

**Figure 7:**
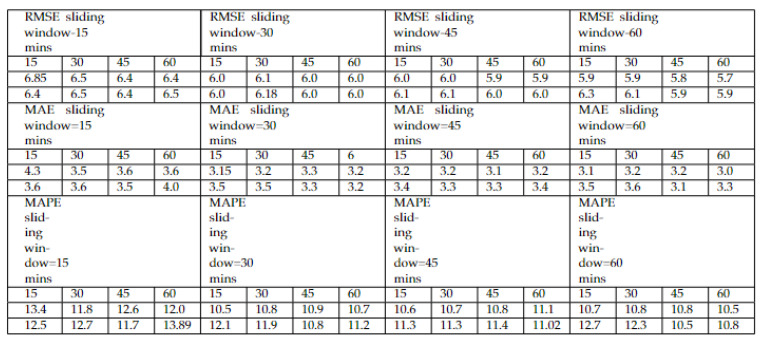
Evaluation metrics of prediction on test data.

Table 4 elaborates the three performance metrics, i.e., *RMSE*, *MAE*, and *MAPE*, with respect to lowest *RMSE*-generated model, i.e., hybrid LSTM–GRU and GRU–LSTM models. In Figure 7, the prediction horizon indicates the capturing of data intervals such as 15, 30, 45, and 60 min. This helps in analyzing the time resolution impact on the model training. As we increased the sliding window, our test data performance improves, which proves a direct proportion of sliding window with the performance metrics. Table 4 contains the different performance metrics behavior on multiple individuals as well as hybrid deep learning models. As per *RMSE* and *MAE* evaluation metrics, hybrid LSTM–GRU produced the lowest *RMSE* of 4.5, *MAE* of 2.03, and *MAPE* of 6.67% being the lowest error.

## 5. Conclusions

This paper utilizes the speed performance index (*SPI*) as the road network state evaluation indicator. We integrated heterogeneous data, i.e., traffic, GPS, weather, special condition, and OSM obtained from a variety of sensors and services. We analyzed the behavior of transportation data sources with the help of different machine and deep learning algorithms. The LSTM–GRU model proved to be the most effective hybrid model among all time series deep learning and classical machine learning models with a net *RMSE* yield of 4.5. In the future, we plan to automatically label the classes using fuzzy logic and k-means clustering, followed by analyzing the results automatically by using optimization of hyper parameters and statistical models.

## Figures and Tables

**Table 1:** Hybrid Feature Space.

Nature of Attributes	Data Type
Day	integer
Hour	integer
Startnode	integer
Endnode	integer
aggminutes	15 min time interval
Weather	char
maxspeed-real	integer
aggSpeed	integer
Holiday	boolean

**Table 2:** Hyperparameters configuration for XGBoost, ANN, KNN, MLP, and Hybrid LSTM–GRU.

Model	Hyperparameters	Values
XGBOOST	objective	linear
	n-estimators	4000
ANN	input dimension	10
	activation function	relu
	loss function	*RMSE*
	optimizere	adam
	epoch	100
	batch-size	512
KNN	K	20
	loss	*RMSE*
MLP	activation function	relu
	loss function	*RMSE*
	hidden-layer-size	100
	optimizer	SGD
	learning rate	0.001
**LSTM-GRU**	Batch Size	512
	Learning Rate	0.001
	No of epochs	10
	No of Hidden Layers	04
	Hidden Units	256
	Dropout Ratio	0.2
	Activation Function	tanh
	Output-Units	1
	Output-Type	Single Label
	Output-Layer-Activation-Function	linear
	Optimizer	Adam
	Loss Function	mean squared error

**Table 3:** Feature selection through correlation feature selection technique.

Features	Scores
start−node	**14,218.665540**
end−node	**52.788980**
way−id	**19,974.123749**
day	**487.561578**
hour	**24,238.112125**
agg−minutes	**0.380622**
quarter	**25.339737**
holiday	**620.959742**
peakhour	**9876.836458**
maxspeed−real	**3,227,692.593161**

**Table 4:** Performance metrics of deep learning models.

Model	*RMSE*	*MAE*	*MAPE*
LSTM	4.86	2.13	6.95
GRU	5.05	2.29	7.7
CNN	30.3	25.96	64.10
LSTM−CNN	4.7	23.9	7.9
CNN−LSTM	5.89	3.53	11.47
GRU−LSTM	4.6	2.08	6.85
LSTM−GRU	**4.5**	**2.03**	**6.67**
CNN−GRU	5.1	2.4	8.4
GRU−CNN	7.8	4.5	14.6

## Data Availability

The data presented in this study are available upon request from the corresponding author.
